# Targeting the Notch Signaling Pathway to Treat Atherosclerosis

**DOI:** 10.3390/cells15161463

**Published:** 2026-08-15

**Authors:** Alexander Blagov, Daria Borodko, Ulyana Rozhkova, Stanislav Antonov, Aleksandra Utkina, Tatiana Kovyanova

**Affiliations:** 1 Laboratory of Molecular Genetic Modeling of Inflammaging, Institute of General Pathology and Pathophysiology, 8, Baltiiskaya Street, 125315 Moscow, Russia; 2 Institute for Atherosclerosis Research, 4-1-207, Osennyaya Street, 121609 Moscow, Russia; 3 Department of Commodity Expertise and Customs Business, Plekhanov Russian University of Economics, 36, Stremyanny Lane, 115054 Moscow, Russia

**Keywords:** notch signaling, atherosclerosis, endothelial dysfunction, vascular smooth muscle cells, macrophage polarization, Jagged1, delta-like ligand 4, gamma-secretase inhibitor, vascular inflammation, therapeutic target

## Abstract

Atherosclerosis remains the principal underlying cause of myocardial infarction, ischemic stroke and peripheral artery disease, and its progression reflects a complex interplay between lipid accumulation, endothelial dysfunction, chronic vascular inflammation and maladaptive remodeling of the arterial wall. The Notch signaling pathway, an evolutionarily conserved juxtacrine communication system, has emerged as a central regulator of every cell type implicated in atherogenesis, including endothelial cells, vascular smooth muscle cells, monocytes/macrophages and T lymphocytes. Depending on the receptor–ligand pairing, the hemodynamic context and the cellular compartment involved, Notch signaling can be either atheroprotective or atherogenic, a duality that has complicated efforts to translate mechanistic insight into therapy. This review summarizes current knowledge of the molecular architecture of the Notch pathway in the vasculature, dissects its cell type-specific and stage-specific contributions to atherosclerotic plaque initiation, progression, calcification and destabilization, and critically appraises pharmacological strategies designed to modulate Notch activity, including γ-secretase inhibitors, ligand- and receptor-directed monoclonal antibodies, soluble decoy receptors, microRNA-based approaches and drug repurposing strategies such as statins. Particular attention is paid to the cardiovascular toxicities that have emerged from oncology trials of Notch pathway inhibitors, which illustrate both the pharmacological tractability and the narrow therapeutic window of this pathway. We conclude that Notch-directed therapy for atherosclerosis is mechanistically well justified but will require cell type-selective and context-selective delivery strategies to be clinically viable.

## 1. Introduction

Atherosclerosis is a chronic disease of medium- and large-sized arteries characterized by the sub-endothelial retention of apolipoprotein B-containing lipoproteins, recruitment and differentiation of monocytes into lipid-laden macrophages, proliferation and phenotypic switching of vascular smooth muscle cells (VSMCs), and progressive fibrous and, in advanced lesions, calcific remodeling of the vessel wall [1]. Phenotypic switching refers to the capacity of medial VSMCs, which under physiological conditions are quiescent, non-proliferative and specialized for contraction, to lose this contractile identity and convert to a proliferative, migratory, extracellular matrix-secreting “synthetic” phenotype in response to injury, inflammatory cytokines and altered mechanical or lipid cues in the vessel wall; these dedifferentiated VSMCs migrate from the media into the intima, where they contribute directly to plaque growth and, depending on context, can further transdifferentiate toward macrophage-like, osteochondrogenic or mesenchymal stem cell-like states that shape both plaque expansion and fibrous cap stability [2]. Atherosclerosis is a multifactorial disease driven by the interaction of non-modifiable risk factors, including age, male sex and family history/genetic predisposition, with modifiable risk factors such as hypertension, dyslipidemia, cigarette smoking, diabetes mellitus, obesity and physical inactivity, all of which converge on the endothelium and the vessel wall to initiate and accelerate the pathological process described above; the clinical consequences of the resulting arterial plaque burden include myocardial infarction, ischemic stroke, peripheral artery disease and sudden cardiac death, which together account for the leading share of global cardiovascular mortality [3]. Despite considerable progress in lipid-lowering and anti-inflammatory pharmacotherapy, atherosclerotic cardiovascular disease continues to account for a very large share of global mortality, which has sustained interest in signaling pathways that coordinate the behavior of the several cell types that jointly build the atherosclerotic plaque.

The Notch pathway is one of a small number of signaling systems that is reused throughout metazoan development to control cell fate decisions [4], and canonical Notch signal transduction proceeds through a well-defined sequence of proteolytic and transcriptional events [5]. It has been co-opted by the adult vasculature to regulate arterial identity, angiogenic sprouting, endothelial quiescence and smooth muscle differentiation [6]. Critically, endothelial Notch signaling also plays a pivotal role in angiocrine signaling, whereby ligand- and receptor-dependent Notch activity in the endothelium couples vessel growth and quiescence to organ-specific paracrine outputs that instruct the behavior of neighboring parenchymal, stromal and immune cells; this angiocrine dimension of endothelial Notch biology has been comparatively underemphasized in the atherosclerosis literature relative to its established roles in vascular morphogenesis and is discussed further below [7]. Germline studies in mice have shown that Notch pathway components are essential for cardiac valve and outflow tract morphogenesis [8]. Because Notch signaling depends on direct membrane contact between a receptor-bearing cell and a ligand-bearing neighbor, it is uniquely suited to encode the local, cell-to-cell information exchange that governs the heterocellular architecture of the artery wall—an architecture that is fundamentally disrupted in atherosclerosis.

Human genetics provides some of the strongest evidence that dysregulated Notch signaling produces overt disease phenotypes. Somatic activating mutations in NOTCH1 are found in more than half of human T-cell acute lymphoblastic leukemias [9], establishing the general principle that quantitative dysregulation of Notch signal strength, and not merely its presence or absence, can be pathogenic. In the cardiovascular system specifically, heterozygous loss-of-function NOTCH1 mutations cause a spectrum of developmental aortic valve anomalies together with severe, adult-onset valve calcification in autosomal-dominant human pedigrees [10], dominant cysteine-altering mutations in NOTCH3 cause cerebral autosomal-dominant arteriopathy with subcortical infarcts and leukoencephalopathy (CADASIL), a stroke- and dementia-causing small-vessel disease [11], and mutations in the ligand JAG1 cause Alagille syndrome, a multisystem disorder with prominent cardiovascular involvement [12]. Consistent with these human phenotypes, genetic studies in mice show that Notch3 is required for arterial identity and postnatal maturation of VSMCs [13], and that the ligand DLL4 is dosage-sensitive during artery development, such that even heterozygous loss causes embryonic vascular defects and lethality [14,15]; a broader survey of Notch pathway mutants confirmed that haploinsufficiency at several nodes of the pathway produces arteriovenous malformations [16]. These monogenic and developmental phenotypes demonstrate unambiguously that Notch dosage is critical for the structural integrity of arteries.

Over the past two decades, an expanding body of experimental work has shown that Notch signaling is also reactivated, in a receptor- and context-dependent manner, in the adult atherosclerotic vessel wall [17,18]. Somewhat counterintuitively, activation of the same core pathway can be atheroprotective when engaged by NOTCH1 in endothelial cells exposed to high, unidirectional shear stress, and atherogenic when engaged by JAG1–NOTCH4 or DLL4 in endothelial cells exposed to low, oscillatory shear stress, or when engaged in monocyte-derived macrophages and VSMCs [19]. This activation of the pathway in atherosclerosis is not merely inferred from review-level synthesis: direct primary evidence includes immunohistochemical demonstration of Notch pathway component upregulation in luminal endothelium overlying human and murine atherosclerotic lesions, and colocalization of activated Notch1 with CD68+ macrophages that is further increased in human vulnerable plaques, findings that have been extended by macrophage-specific genetic loss-of-function studies confirming a causal, pro-atherogenic role for Notch1 activation in this compartment [20]. This apparent paradox—the same pathway acting as both a guardian and a driver of vascular disease—is the central challenge that any Notch-directed therapeutic strategy for atherosclerosis must resolve. In this review, we first summarize the molecular logic of canonical and non-canonical Notch signaling, then examine its roles in the principal cellular participants in atherogenesis, and finally evaluate the pharmacological and biological strategies currently available, or in development, to modulate Notch signaling for the treatment of atherosclerosis, including a critical discussion of the cardiovascular safety signals that have emerged from clinical experience with Notch inhibitors developed for oncology indications.

## 2. Molecular Architecture of the Notch Signaling Pathway

### 2.1. Canonical Notch Signaling

Mammals express four Notch receptors (NOTCH1–4) and five canonical ligands of the DSL (Delta/Serrate/Lag-2) family: Delta-like ligand 1, 3 and 4 (DLL1, DLL3, DLL4) and Jagged1 and Jagged2 (JAG1, JAG2) [4,5]. Both receptors and ligands are type I single-pass transmembrane proteins, so that productive signaling requires direct contact between the signal-sending and signal-receiving cell. Following synthesis, the Notch receptor is cleaved once in the trans-Golgi network by a furin-like convertase (S1 cleavage), yielding a heterodimeric receptor displayed at the plasma membrane. Engagement of a ligand on a neighboring cell exerts a mechanical pulling force on the receptor extracellular domain, unmasking a negative regulatory region and permitting a second cleavage (S2) by an ADAM-family metalloprotease. This step generates a membrane-tethered stub that is then subjected to intramembrane proteolysis (S3 cleavage) by the γ-secretase complex, releasing the Notch intracellular domain (NICD) into the cytoplasm [5]. The NICD translocates to the nucleus, where it associates with the DNA-binding protein RBP-J (also known as CSL or CBF1), displacing a co-repressor complex and recruiting the coactivator Mastermind to activate transcription of target genes, most notably the basic helix–loop–helix repressors of the Hes and Hey families [5]. Consistent with the importance of tightly regulated proteolytic release of the NICD, mice doubly deficient for the Notch target genes Hey1 and Hey2 develop severe congenital cardiac defects due to impaired epithelial-to-mesenchymal transition during valve formation [21]. Because ligand-induced receptor cleavage is essentially irreversible and each Notch molecule signals only once, the pathway lacks the second-messenger amplification typical of many other signaling systems; instead, signal strength and duration are encoded by the number of productive receptor–ligand engagements, the local concentration and glycosylation state of ligands, and the specific receptor–ligand pairing, all of which can differ markedly between vascular cell types and hemodynamic environments.

### 2.2. Non-Canonical Notch Signaling and Ligand-Dependent Bias

In addition to the canonical RBP-J-dependent pathway, Notch receptors and ligands participate in non-canonical, transcription-independent signaling events. Membrane-tethered NOTCH1 fragments generated by S2 cleavage can associate with adherens junction components, including VE-cadherin and the Rho-GEF Trio, to regulate junctional stability independently of NICD release and nuclear signaling [22]. Ligands, too, can act outside the canonical receptor-activation cascade: Jagged1 has recently been shown to function as a Notch-independent mechanotransducer, engaging VEGFR2/ERK signaling through Src-dependent mechanisms in endothelial cells exposed to fluid shear stress, in a manner that does not require canonical Notch cleavage [23]. Furthermore, ligands presented in cis (on the same cell as the receptor) generally inhibit rather than activate Notch signaling in trans, a phenomenon termed cis-inhibition, which allows a single cell that co-expresses both a Notch receptor and a Notch ligand to modulate its own responsiveness to signals from neighboring cells. In practical terms, this means that a ligand molecule engages Notch receptors productively only when it is presented by a neighboring cell (in trans); when the same ligand is instead expressed on the surface of the receptor-bearing cell itself (in cis), it binds and sequesters the receptor without triggering the proteolytic activation cascade, thereby acting as a competitive brake rather than an activator. Because most vascular cells co-express several Notch receptors and ligands simultaneously, the net signaling output of any given cell is therefore not simply set by which ligands and receptors are present, but by the balance between cis-inhibitory and trans-activating interactions, a property that allows cells to sharpen boundaries between distinct signaling states and that adds a further, receptor-intrinsic layer of context-dependence to Notch outputs in the vessel wall. Mechanistically, this asymmetry arises because trans-activation and cis-inhibition rely on different physical requirements for productive receptor engagement. Trans-activation depends on the mechanical pulling force generated when a ligand anchored to the plasma membrane of a neighboring cell is endocytosed together with the bound Notch ectodomain, a tugging step that is only possible across a cell–cell junction and that mechanically exposes the S2 cleavage site to ADAM-family proteases; a ligand and receptor confined to the same cell membrane cannot generate this trans-cellular pulling force. Instead, single-cell imaging and mathematical modeling of the Notch-Delta system have shown that co-expressed receptor and ligand on one cell rapidly form a stable, mutually inactivating cis-complex that sequesters both molecules away from the pool available for productive trans-engagement, so that cis-pairing acts as a fast, cell-autonomous “off-switch” that effectively titrates a cell’s own Notch responsiveness rather than triggering its proteolytic activation cascade [24]. These non-canonical and cis-regulatory features substantially expand the combinatorial repertoire of the Notch system and help explain why manipulating a single receptor or ligand can produce markedly different, sometimes opposite, phenotypes depending on the cellular and mechanical context.

### 2.3. Post-Translational Regulation: Glycosylation and Ligand Affinity Tuning

The affinity and outcome of a given Notch receptor–ligand interaction is further tuned by glycosylation of the receptor extracellular domain. Fringe family glycosyltransferases add N-acetylglucosamine residues to O-fucose modifications on specific epidermal growth factor-like repeats of the Notch ectodomain; mass-spectrometric mapping has shown that modification at distinct repeats potentiates Delta-like ligand binding while simultaneously inhibiting Jagged1-induced activation of the same receptor [25]. Of the three mammalian Fringe paralogues, Lunatic Fringe exerts a dominant effect over Manic and Radical Fringe on NOTCH1 signaling output, such that the relative expression of Lunatic Fringe in a given vascular cell can determine whether that cell preferentially responds to Delta-like or Jagged ligands [26]. This Fringe-dependent switch in ligand preference was first established mechanistically by Benedito and colleagues, who showed in the developing retinal vasculature that Jagged1 is a comparatively weak NOTCH1 agonist that behaves as a potent, Fringe-modulated proangiogenic antagonist of Dll4-Notch signaling, so that the balance between these two ligands, rather than the presence of either alone, dictates tip- versus stalk-cell selection and vascular sprouting; this same Fringe-gated Dll4/Jagged1 antagonism is directly relevant to the differential, sometimes opposing, roles of these two ligands in the endothelium of the atherosclerotic vessel wall discussed in Section 3.1 [27]. Because vascular cells differentially express Fringe enzymes depending on developmental stage, flow environment and disease state, the glycosylation status of the endothelial or smooth muscle Notch receptor pool represents an additional, cell-intrinsic layer of regulation that can bias the outcome of ligand engagement without any change in the absolute abundance of either ligand class. The three Fringe paralogues show distinct, non-redundant expression patterns across the vessel wall. Manic Fringe is constitutively expressed by vascular endothelial cells of arteries, veins and capillaries and is further upregulated in pathologically remodeling, hyperangiogenic endothelium, where its loss impairs endothelial proliferation, migration and sprouting, indicating a specific endothelial requirement for this paralogue [28]. Radical Fringe, in contrast, is comparatively enriched in mural cells, and its expression rises markedly in senescent human pericytes, where it modulates the glycosylation, turnover and signaling output of NOTCH3, including of the CADASIL-associated NOTCH3 mutant proteins that accumulate in degenerating vascular smooth muscle and pericytes, pointing to an age- and mural cell-specific role for this paralogue that is distinct from Manic Fringe’s endothelial function [29]. This glycosylation-based bias mechanism is not yet well characterized in the atherosclerotic vessel wall specifically, but by analogy with its role in other tissues, it offers a further, largely unexploited, layer at which pharmacological or gene-based intervention could in principle be used to favor atheroprotective over atherogenic Notch signaling outputs. Notch pathway components relevant to vascular biology and atherosclerosis are summarized in Table 1.

## 3. Notch Signaling in the Principal Cell Types of the Atherosclerotic Vessel Wall

### 3.1. Endothelial Cells: A Mechanosensitive Switch Between Atheroprotection and Atherogenesis

The endothelium is the first vascular compartment to sense hemodynamic forces, and atherosclerotic lesions form preferentially at arterial branch points and curvatures where blood flow is disturbed and oscillatory, rather than in regions of high, unidirectional laminar shear stress [30]. Endothelial NOTCH1 behaves as a bona fide mechanosensor: canonical NOTCH1 activity scales with the magnitude of fluid shear stress, is required for the maintenance of endothelial junctional integrity, cell elongation and suppression of proliferation under laminar flow, and its endothelial-specific deletion increases hypercholesterolemia-induced atherosclerosis in the murine aorta, establishing NOTCH1 as atheroprotective in this context [30]. Mechanistically, NOTCH1 mechanotransduction appears to combine a ligand-dependent and a ligand-independent component rather than acting as a purely intrinsic force sensor. On one hand, comparison of blood and lymphatic endothelial cells that express comparable levels of NOTCH1, DLL4, JAG1 and VE-cadherin showed that laminar shear stress activates NOTCH1 proteolysis only in the blood vascular subtype, indicating that flow-responsive activation is not explained simply by receptor-ligand co-expression but requires a cell type-specific configuration of the DLL4-NOTCH1 complex at the junction, consistent with force-dependent unmasking of the ADAM10 cleavage site during ligand engagement under flow. On the other hand, shear stress also engages the mechanosensitive ion channel Piezo1, and the resulting calcium influx increases ADAM10 activity, providing a second, receptor-extrinsic route by which mechanical force can amplify NOTCH1 proteolysis independently of any single ligand; downstream, the liberated NOTCH1 intracellular domain associates with VE-cadherin to stabilize adherens junctions, linking the mechanosensing step directly to the junctional-integrity phenotype described above. NOTCH1 is therefore best understood as a mechanosensor whose activation is jointly gated by flow-dependent ligand engagement and by a parallel, ADAM10-sensitizing Piezo1-calcium axis, rather than by either mechanism alone [52]. Complementary human tissue data support this conclusion: NOTCH1 is constitutively expressed at high levels in adult arterial endothelium relative to smooth muscle, and its expression and downstream signaling are markedly suppressed by a high-fat diet and by circulating inflammatory lipids, an effect that antagonizes endothelial activation, so that mice with only one functional Notch1 allele in the endothelium develop more extensive diet-induced atherosclerosis [53]. The suppression of endothelial NOTCH1 by hyperlipidemia is not merely a correlative diet effect but reflects a direct, ligand-independent transcriptional mechanism: specific oxidized phospholipid species that accumulate under high-fat feeding rapidly repress NOTCH1 and JAG1 expression through activation of the transcription factor STAT3, and this Notch suppression is reversed when circulating cholesterol is lowered, indicating that circulating oxidized lipids actively silence the atheroprotective Notch1 axis rather than NOTCH1 loss being a secondary consequence of established atherosclerosis [53]. More broadly, native atherosusceptible and atheroresistant regions of the arterial tree show reproducible differences in endothelial gene expression and phenotype that mirror the effects of disturbed versus laminar shear stress applied experimentally, and Notch pathway genes feature prominently among the flow-responsive transcripts that distinguish these regions [54,55].

In striking contrast to this atheroprotective role of NOTCH1, the ligand JAG1 together with the receptor NOTCH4 is preferentially expressed at atherosusceptible sites exposed to low, oscillatory shear stress, and endothelial-specific deletion of Jag1 reduces atherosclerotic lesion burden at these sites in murine and porcine arteries, indicating that JAG1–NOTCH4 signaling is pro-atherogenic [34]. Single-cell transcriptomic analysis of Jag1-deficient endothelium suggests that JAG1 suppresses reparative, proliferative and migratory endothelial subsets that would otherwise counteract disturbed-flow-induced dysfunction, providing a plausible mechanism for the pro-atherogenic action of this ligand [34]. Mechanistically, this reflects a shift in endothelial cell-state composition rather than a uniform toxic effect on all endothelial cells: JAG1-NOTCH4 signaling represses a specific transcriptional program associated with proliferation, migration and repair, so that in its absence disturbed-flow endothelium is enriched for these reparative subsets, whereas active JAG1-NOTCH4 signaling depletes them and leaves the endothelium dominated by cell states that are less able to counteract flow-induced injury, adhesion-molecule induction and barrier dysfunction; this loss of reparative capacity, rather than a direct pro-inflammatory transcriptional program analogous to that driven by NOTCH1, is proposed as the principal mechanism by which JAG1-NOTCH4 signaling enhances atherosclerosis susceptibility at sites of disturbed flow [34]. Complementary zebrafish experiments show that the absence of blood flow itself upregulates the ligand Dll4 in the developing vasculature, indicating that flow tonically suppresses certain Notch ligands in vivo and that loss of flow, as occurs at recirculating or stagnant sites in diseased arteries, could remove this brake [56]. Because JAG1 can act as a cis-inhibitor of NOTCH1—that is, it can bind and neutralize NOTCH1 when the two are expressed on the same endothelial cell, rather than activating it as it would from a neighboring cell—elevated JAG1 expression at disturbed-flow regions may simultaneously suppress atheroprotective NOTCH1 signaling while engaging the distinct, atherogenic NOTCH4 receptor pool, offering one explanation for how the same pathway components can produce opposite regional outcomes within a single vessel [23].

Independent of its role as a mechanosensor, Notch signaling regulates several features of endothelial dysfunction that are central to atherogenesis. Enforced NOTCH1 activation—achieved experimentally by overexpressing the constitutively active Notch1 intracellular domain, the same gain-of-function approach used in the analogous tumor-endothelium studies discussed above—in cultured and in vivo endothelium upregulates pro-inflammatory cytokines (IL-6, IL-8, IL-1α), induces a senescent phenotype, and correlates with coronary artery disease risk in genotyped patient cohorts, while Notch pathway components are selectively upregulated in the luminal endothelium overlying atherosclerotic lesions in both mouse and human aortas [57]. Inflammatory stimuli such as TNF-α and IL-1β further dysregulate endothelial Notch signaling, in particular by downregulating NOTCH2 and NOTCH4, contributing to endothelial barrier dysfunction and adhesion molecule expression [31]. Endothelial Jagged1–RBP-J signaling has also been shown to promote recruitment of inflammatory leukocytes to the vessel wall and to exacerbate atherosclerosis in mouse models [36], and in a rat cardiac allograft model of transplant arteriosclerosis, TNF-mediated downregulation of Notch4 impaired endothelial quiescence and provoked endothelial cell activation and apoptosis, an effect that could be reproduced by direct Notch4 silencing and that was linked mechanistically to loss of RBP-J-dependent survival signaling [35,58]. Conversely, forced activation of a Notch1–Jagged1 autocrine circuit in cultured endothelial cells is sufficient to induce VCAM1 expression on its own and to amplify IL-1β-driven VCAM1 induction, directly linking Notch1 gain-of-function to a classical adhesion-molecule readout of endothelial activation [59]. The pathophysiological consequences of this circuit are illustrated most completely outside the atherosclerosis literature itself, in tumor endothelium, where sustained Notch1 intracellular domain activity induces a senescence-like endothelial phenotype together with VCAM1 upregulation, thereby promoting neutrophil infiltration and leukocyte–endothelial adhesion; because tumor-associated endothelial Notch1 activation, VCAM1 induction and senescence recapitulate the same triad implicated in atherosclerotic endothelial dysfunction, this work provides an important, complementary mechanistic precedent for the role of endothelial Notch1 gain-of-function in driving vascular inflammation and leukocyte recruitment within the plaque [60]. Endothelial microRNA regulation adds a further layer to this network: shear-responsive miR-126-5p maintains endothelial proliferative reserve by suppressing the non-canonical Notch1 inhibitor Dlk1, and mice lacking miR-126-5p show reduced endothelial Notch1 activation and exacerbated atherosclerotic lesion formation, whereas restoring miR-126-5p reduces lesion size, illustrating a distinct, atheroprotective arm of endothelial Notch1 signaling operating through de-repression by a flow-sensitive microRNA [61]. Beyond atherosclerosis proper, endothelial NOTCH1 is required for normal postnatal angiogenesis and ischemia-induced neovascularization [62], and its proteolytic activation in this setting is promoted by the cysteine protease cathepsin K, which increases NICD generation and downstream Hes/Hey target gene expression in ischemic tissue, again illustrating that the same receptor supports reparative vascular functions in some contexts even as its dysregulation promotes disease in others [63]. Beyond its role in shaping lesion-related inflammation and remodeling, Notch signaling also plays an active, ongoing role in maintaining the quiescence of the established, non-diseased vasculature, a function that is directly relevant to the safety of pharmacological Notch inhibition discussed in Section 5. Genetic or antibody-mediated inhibition of endothelial Notch signaling in the adult heart impairs endothelial fatty acid transport and provokes pathological angiogenesis, metabolic remodeling and heart failure, demonstrating that tonic Notch activity is required to actively restrain angiogenesis and preserve normal cardiac vascular and metabolic homeostasis rather than being dispensable outside disease states [64]. Consistent with this quiescence-maintaining function, forced endothelial expression of constitutively active Notch4 is sufficient to produce arteriovenous malformations in multiple organs, including the liver, uterus and skin, a phenotype that regresses upon withdrawal of the activating stimulus, underscoring that the adult vasculature depends on continuously balanced, rather than simply present or absent, Notch signaling to maintain normal vessel architecture [65].

### 3.2. Vascular Smooth Muscle Cells: NOTCH3 and the Contractile-to-Synthetic Phenotypic Switch

Among the four Notch receptors, NOTCH3 shows the most restricted and abundant expression in adult vascular smooth muscle, where it is required for normal arterial mural cell differentiation, postnatal maturation and survival [13]. The physiological importance of NOTCH3 in VSMCs is illustrated most dramatically by CADASIL, in which stereotyped cysteine-altering NOTCH3 mutations cause pathological accumulation of the receptor extracellular domain around degenerating smooth muscle cells of small cerebral arteries [32], a process that has been reproduced in transgenic mice expressing mutant NOTCH3 and that recapitulates the granular osmiophilic material and progressive arteriopathy seen in patients [66]. Although CADASIL is a disease of small cerebral arterioles rather than of large-vessel atherosclerosis, it provides unambiguous proof-of-principle that dysregulated NOTCH3 signaling, whether through gain- or loss-of-function mechanisms, is sufficient to produce a severe occlusive arteriopathy in humans. Under physiological conditions, NOTCH3 expression in mural cells is itself maintained by an autoregulatory loop that requires endothelial-expressed JAG1, coupling the endothelial and smooth muscle compartments of the vessel wall [37], and reciprocal signaling through JAG1 promotes NOTCH3-dependent contractile differentiation of human coronary artery smooth muscle cells in three-dimensional coculture systems [67,68], underscoring that VSMC phenotype in the mature vessel wall is actively maintained by ongoing paracrine Notch signaling from the overlying endothelium rather than being fixed during development alone. In keeping with this, global loss of Notch3 is further associated with defective angiogenesis and impaired mural cell investment of new vessels [69], broadening the phenotypic consequences of NOTCH3 loss beyond large-vessel structural integrity to microvascular remodeling more generally.

In the context of atherosclerosis and neointimal remodeling, Notch signaling—engaged through NOTCH1 as well as NOTCH3—drives the transition of VSMCs from a quiescent, contractile phenotype toward a proliferative, synthetic and migratory phenotype. Pharmacological inhibition of γ-secretase with the compound DAPT restores expression of contractile marker genes, suppresses VSMC proliferation, and reduces intimal hyperplasia in a rat vein-graft model of restenosis [50], and, in models of diabetic microangiopathy, dysregulated Jagged1–Notch1 signaling has similarly been linked to abnormal, dysfunctional neovascularization and delayed angiogenic repair [70]. These findings position Notch signaling as a convergence point for multiple drivers of vascular remodeling, spanning mechanical injury, restenosis and metabolic disease.

Notch signaling additionally participates in the osteogenic trans-differentiation of VSMCs that underlies vascular calcification, a hallmark of advanced atherosclerotic plaques and an independent predictor of cardiovascular events. Direct activation of Notch signaling in human aortic smooth muscle cells induces the osteogenic master regulator Msx2 and subsequent alkaline phosphatase activity and matrix mineralization in a manner dependent on Notch–RBP-J signaling [49], and Notch3 activation downstream of hepatocyte growth factor/c-Met signaling similarly promotes VSMC osteogenic differentiation in vitro [33]. microRNA-34a, whose expression rises with vascular aging and in response to atherogenic and uremic stimuli, promotes VSMC senescence and a senescence-associated secretory phenotype by downregulating SIRT1, and this senescence program favors the osteochondrogenic transdifferentiation of VSMCs and medial calcification [71]; mechanistically, miR-34a has been shown to promote calcification specifically through downregulation of SIRT1 and the receptor tyrosine kinase Axl [72]. A parallel, valve-specific literature shows that Notch1 normally represses osteogenic gene programs in aortic valve interstitial cells [73], that mice haploinsufficient for the Notch effector RBPJK develop diet-induced calcific aortic valve disease with macrophage infiltration and pro-osteogenic signaling [74], that a disease-associated Notch1 mutation promotes valvular calcification through enhanced myofibroblast mechanotransduction [75], and that pharmacological targeting of the mechanosensitive protein cadherin-11 can prevent Notch1-mediated calcific aortic valve disease in animal models [76]—collectively illustrating a coherent, receptor-specific link between disrupted Notch signaling, altered mechanotransduction and ectopic osteogenic differentiation that spans both the valvular and arterial vasculature.

### 3.3. Macrophages and the Innate Immune Response

Monocyte-derived macrophages are the dominant immune cell population within atherosclerotic plaques, where they take up modified lipoproteins to become foam cells, secrete pro-inflammatory mediators, and, in advanced lesions, undergo apoptosis and defective efferocytosis to form the necrotic lipid core that predisposes to plaque rupture [1]. Notch signaling was first shown to be functionally upregulated following macrophage activation, where it modulates gene expression programs relevant to antigen presentation and cytotoxic activity [45], and canonical Notch–RBP-J signaling has subsequently been shown to amplify Toll-like receptor 4 (TLR4)-induced expression of classically activated, pro-inflammatory M1-like macrophage genes by controlling translation of the transcription factor IRF8 [46]. Consistent with a pro-inflammatory, M1-like-promoting role, forced Notch intracellular domain expression in macrophages upregulates M1-like-associated genes and increases tumor cell cytotoxicity in antitumor immune models [77], whereas myeloid-specific disruption of RBP-J favors an alternatively activated, M2-like phenotype in models of chitin-induced M2-like polarization [47], although RBP-J also supports selected components of the M2-like transcriptional program in the liver, where its myeloid-specific loss ameliorates hepatic fibrosis by attenuating inflammation [48]—indicating that the relationship between Notch signaling and macrophage polarity is more nuanced than a simple M1-like/M2-like binary switch.

In the specific setting of vascular and metabolic disease, endothelial-derived Delta-like ligand 4 was among the first Notch ligands shown to directly induce Notch signaling in macrophages with pro-inflammatory consequences [40], and subsequent work demonstrated that a high-fat, high-cholesterol diet increases Dll4 expression in atheromata and that systemic blockade of Dll4 with a neutralizing antibody attenuates both atherosclerotic lesion formation and the associated metabolic derangements in hyperlipidemic mice, an effect attributed at least in part to reduced macrophage-driven inflammation [41]. Macrophage-derived Dll4 has similarly been shown to promote lesion development in a vein-graft model relevant to bypass graft failure [78], and, at the cellular level, Dll4 signaling through Notch1 selectively impairs the differentiation of blood monocytes into anti-inflammatory M2-like macrophages while promoting their apoptosis, without affecting M1-like differentiation, providing a mechanistic basis for how a single ligand can simultaneously favor pro-inflammatory macrophage programs and actively deplete the reparative, inflammation-resolving macrophage pool within the plaque [42]. At the level of cellular metabolism, Notch signaling has also been shown to reprogram macrophage mitochondrial function toward a pro-inflammatory bioenergetic profile, providing a further, metabolic layer through which Notch sustains the M1-like phenotype through a specific, dual mechanism rather than a generic metabolic shift: the Notch1 intracellular domain is recruited directly to nuclear and mitochondrial genes encoding respiratory chain components, increasing mitochondrial oxidative phosphorylation, while, in parallel, Notch-dependent induction of pyruvate dehydrogenase phosphatase 1 raises pyruvate dehydrogenase activity and glucose flux into the tricarboxylic acid cycle; the resulting increase in oxidative flux elevates mitochondrial reactive oxygen species (mtROS) production, and this mtROS signal feeds back to reinforce transcriptional induction of the same pro-inflammatory gene program, so that Notch couples a transcriptional and a bioenergetic arm into a single, self-amplifying circuit that sustains macrophage activation [79].

**Figure 1:**
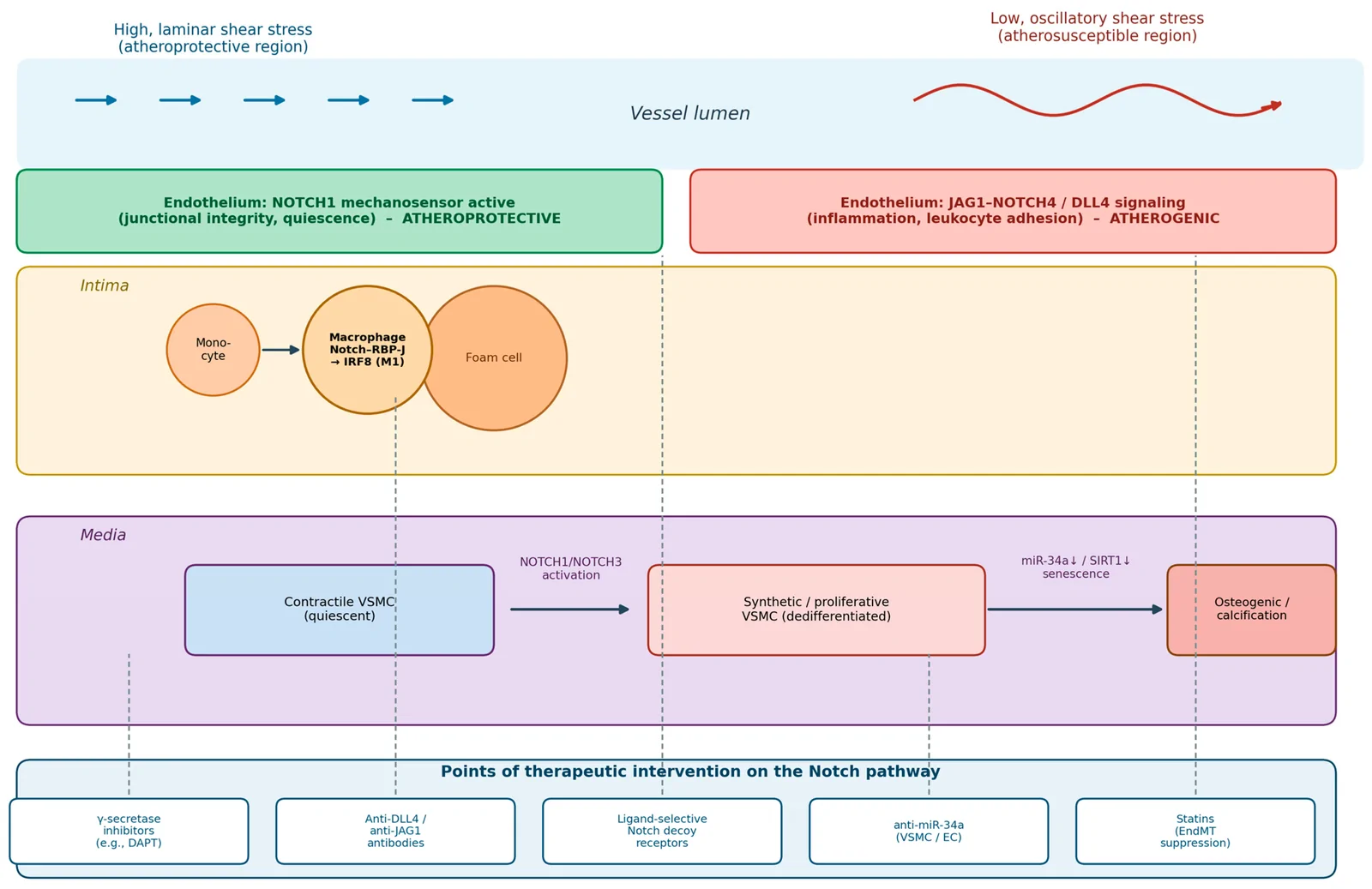
Schematic overview of Notch signaling across the atherosclerotic vessel wall. At sites of high, laminar shear stress, endothelial NOTCH1 preserves junctional integrity and quiescence (atheroprotective, green). At sites of low, oscillatory shear stress, endothelial JAG1–NOTCH4/DLL4 signaling promotes leukocyte adhesion and recruitment (atherogenic, red). Recruited monocytes differentiate into macrophages in which Notch–RBP-J signaling promotes M1-like polarization and foam cell formation. In the media, NOTCH1/NOTCH3 signaling drives the transition of vascular smooth muscle cells (VSMC) from a contractile to a synthetic, proliferative phenotype, and chronic signaling together with miR-34a/SIRT1 dysregulation promotes osteogenic transdifferentiation and calcification. The lower panel indicates the principal points at which pharmacological strategies discussed in Section 5 intersect the pathway: γ-secretase inhibitors, anti-DLL4/anti-JAG1 antibodies, ligand-selective Notch decoy receptors, anti-miR-34a oligonucleotides, and statins acting via suppression of endothelial-to-mesenchymal transition (EndMT).

Beyond the innate compartment, Notch ligands presented by antigen-presenting cells within the plaque shape adaptive immunity. In a foundational study, Delta-family ligands were shown to instruct CD4+ T cells toward the Th1 fate, whereas Jagged-family ligands instruct the alternate Th2 fate, establishing that ligand identity, rather than Notch activation as such, dictates the polarity of the adaptive immune response [38]. It should be noted that this foundational Th1/Th2 finding, like most of the T-cell Notch ligand studies discussed in this section, was established in general immunological or autoimmune disease models rather than in atherosclerosis itself; its relevance to the vessel wall is therefore inferred rather than directly demonstrated. Within atherosclerosis specifically, Th1 polarization is comparatively well established as pro-atherogenic, since interferon-γ-producing Th1 cells and their inducing cytokines aggravate lesion development and plaque instability in multiple loss- and gain-of-function mouse studies, whereas the role of Th2 cells is considerably more controversial, with murine loss-of-function studies for Th2 cytokines yielding inconsistent atheroprotective, neutral or even pro-atherogenic effects depending on the specific cytokine, disease stage and genetic background examined, in contrast to the comparatively consistent picture for Th1 and regulatory T cells [80]. Given this uncertainty, a Delta-family ligand bias that favors Th1 responses would be predicted to be net pro-atherogenic on adaptive-immune grounds, whereas the vascular consequence of a Jagged-family, Th2-skewing bias remains genuinely unresolved and represents an important open question for translating this ligand-specific logic to atherosclerosis. Building on this ligand-specific logic, Notch signaling has subsequently been shown to be required for optimal Th17 differentiation in both mouse and human T cells, with pharmacological Notch blockade ameliorating experimental autoimmune encephalomyelitis, a Th17-dependent inflammatory disease model relevant to chronic vascular inflammation [39]; consistent with the earlier Th1/Th2 paradigm, Jagged1 and Delta1 were further shown to differentially regulate the severity and outcome of this same autoimmune model, with blockade of Jagged1 worsening and blockade of Delta1 improving disease [81]. Notch signaling is not confined to effector T-cell subsets: cell-intrinsic Notch activity in regulatory T cells is required for peripheral immune tolerance, and its disruption in Tregs precipitates spontaneous autoimmune and inflammatory pathology in mice [82], suggesting that the balance of Notch ligands available within the atherosclerotic plaque microenvironment may simultaneously shape effector T-cell polarization and the counter-regulatory activity of Tregs that normally restrains plaque inflammation. Notch signaling mechanisms are depicted on Figure 1.

### 3.4. In Vivo Evidence: Effects of Experimental Notch Manipulation on Atherosclerosis Development

The cell type-specific findings summarized above are drawn from experiments that differ substantially in genetic strategy, species, vascular bed and outcome measure, which can make the net direction of any given manipulation difficult to extract from prose alone. Table 2 therefore consolidates, in one place, the principal in vivo genetic and pharmacological Notch manipulations that have been directly tested in experimental models of atherosclerosis or closely related vascular remodeling, together with their reported effect on lesion or plaque phenotype. As the table illustrates, the direction of effect tracks receptor and cell-type identity rather than simply reflecting “more” or “less” Notch signaling in the vessel as a whole: loss of endothelial NOTCH1 is consistently pro-atherogenic [30,53], whereas loss of endothelial JAG1 [34], blockade of DLL4 [41], or macrophage-restricted loss of NOTCH1 [20] are each atheroprotective, and non-selective pharmacological suppression of the entire pathway with γ-secretase inhibitors is net atheroprotective in the models tested to date [50,51,83], likely because the pro-atherogenic, disturbed-flow-restricted JAG1–NOTCH4 and macrophage arms of the pathway dominate the aggregate outcome under the conditions examined. This consolidated view reinforces the central argument of this review, developed in Section 4 below, that “the Notch pathway” cannot be treated as a single therapeutic target in atherosclerosis.

## 4. Notch Signaling Across the Stages of Atherosclerotic Plaque Development

The multiplicity of cell-type-specific Notch effects described above can be integrated into a stage-based model of atherogenesis. During lesion initiation, disturbed flow at atherosusceptible sites upregulates endothelial JAG1/NOTCH4 signaling, suppresses atheroprotective NOTCH1 signaling, and increases expression of adhesion molecules and inflammatory cytokines that favor monocyte recruitment [30,34,55]. During lesion progression, infiltrating monocytes differentiate into macrophages whose polarization state is shaped by local Delta-like ligand and Jagged1 availability [40,42,46], while resident and migrating VSMCs undergo NOTCH1/NOTCH3-dependent dedifferentiation, proliferation and migration into the intima, expanding the fibrous cap and, in some contexts, contributing to its structural reinforcement [49,50]. In advanced lesions, chronic Notch pathway activity intersects with cellular senescence programs, in particular via the miR-34a/SIRT1 axis, to promote osteochondrogenic transdifferentiation of VSMCs and medial or intimal calcification, a feature associated with both plaque stability in some contexts and abrupt clinical events in others [71,72]. Finally, at the level of the fibrous cap and lipid core, ongoing macrophage Notch–RBP-J signaling sustains a pro-inflammatory, M1-like-skewed macrophage phenotype and actively suppresses the reparative M2-like macrophage pool, impeding efferocytosis and lesion resolution and favoring necrotic core expansion and potentially plaque instability [42,46]. This staged view underscores that “the Notch pathway” is not a single therapeutic target in atherosclerosis but rather a family of related signaling modules whose net effect on disease depends critically on which receptor, which ligand, which cell type and which stage of lesion development is being considered.

## 5. Therapeutic Strategies Targeting the Notch Pathway in Atherosclerosis

Given the pleiotropic, context-dependent roles of Notch signaling in the vessel wall, therapeutic strategies have pursued several distinct levels of intervention: global pharmacological inhibition of the terminal proteolytic step common to all Notch receptors (γ-secretase inhibitors), selective antagonism of individual ligands or receptors using monoclonal antibodies or engineered decoy receptors, indirect modulation through microRNA-based agents, and repurposing of existing cardiovascular drugs, particularly statins, that have off-target effects on Notch-dependent endothelial and smooth muscle phenotypes. Table 1 summarizes the principal molecular components of the pathway relevant to vascular biology, and Table 3 summarizes the therapeutic strategies discussed below together with their stage of development and principal limitations.

### 5.1. Gamma-Secretase Inhibitors

γ-Secretase inhibitors (GSIs) such as DAPT block the terminal S3 cleavage step required for release of the NICD from all four Notch receptors, and were originally developed against other targets before being repurposed as pan-Notch inhibitors in oncology and vascular research; indeed, the discovery that activating NOTCH1 mutations drive a majority of T-cell leukemias provided much of the initial rationale for developing GSIs as targeted anticancer agents [9]. In atherosclerosis-relevant models, the GSI DAPT restores contractile gene expression and suppresses VSMC proliferation in a rat vein-graft model of intimal hyperplasia [50]. More directly, systemic administration of a γ-secretase inhibitor reduces diet-induced atherosclerotic lesion formation in apolipoprotein E-deficient mice, providing in vivo evidence that pan-Notch inhibition can be net atheroprotective in this model, at least over the exposure durations tested [51]. It is important to note that the anti-atherosclerotic effect of pan-Notch GSIs cannot be attributed to Notch blockade alone, since γ-secretase constitutively cleaves dozens of other type I transmembrane substrates that are independently relevant to vascular and inflammatory biology, including N- and E-cadherin, whose cleavage disrupts endothelial and smooth muscle cell–cell adhesion; CD44, a receptor implicated in leukocyte adhesion and macrophage foam-cell behavior; the receptor tyrosine kinase ErbB4; and the low-density lipoprotein receptor-related protein (LRP), which participates directly in lipoprotein handling by the vessel wall. Consequently, part of the atheroprotective effect attributed to GSIs in ApoE-deficient mice and vein-graft models may reflect concurrent inhibition of these non-Notch substrates rather than Notch blockade per se, a caveat that is difficult to exclude with pan-Notch inhibitors and that further motivates the shift toward ligand- and receptor-selective strategies discussed below [90]. However, because γ-secretase also cleaves numerous other type I transmembrane substrates besides Notch receptors, and because pan-Notch blockade indiscriminately silences both atheroprotective (NOTCH1) and atherogenic (NOTCH4, JAG1-dependent) signaling, GSIs carry an unfavorable therapeutic index for chronic cardiovascular use; class-wide toxicities related to Notch-dependent differentiation programs in the gut and lymphoid system have also limited their systemic use in oncology, reinforcing that pan-pathway inhibition is unlikely to be an acceptable long-term strategy for a chronic disease such as atherosclerosis.

### 5.2. Ligand- and Receptor-Directed Monoclonal Antibodies

A more selective approach exploits monoclonal antibodies directed against individual Notch ligands or receptors, an approach extensively developed in oncology to block tumor angiogenesis and cancer stem cell maintenance. Antibodies against Delta-like ligand 4, such as demcizumab, have been evaluated in multiple phase I and phase Ib oncology trials in combination with cytotoxic chemotherapy [43,44]. These trials are informative for cardiovascular pharmacology chiefly because of the adverse events they revealed: dose- and duration-dependent cardiopulmonary toxicity, including grade 3 hypertension, pulmonary hypertension and, in a subset of patients receiving prolonged high-dose therapy, congestive heart failure that was reversible upon drug discontinuation in most cases [44]. A systematic analysis of clinical trials of DLL4-inhibiting antibodies confirmed pulmonary hypertension as a class-associated complication occurring independently of left ventricular dysfunction, implicating disordered angiogenesis and vascular remodeling secondary to sustained DLL4/NOTCH1 blockade in the pulmonary circulation [85]. A parallel phase Ib trial combining demcizumab with paclitaxel in platinum-resistant ovarian cancer similarly documented cardiopulmonary adverse events consistent with this class effect [84]. These findings, while derived from an oncology population receiving substantially higher and more sustained receptor blockade than would likely be used for an atherosclerosis indication, provide a cautionary, real-world demonstration that pharmacological inhibition of the DLL4–NOTCH axis has a genuine and clinically important cardiovascular liability, and they argue for very careful dose-finding, intermittent dosing schedules, or vascular bed-restricted delivery if anti-DLL4 or anti-Jagged1 antibodies are ever to be considered for atherosclerosis. Antibodies selectively targeting NOTCH2/NOTCH3, such as tarextumab, illustrate ongoing efforts within oncology to narrow the toxicity window of Notch pathway antibodies by sparing NOTCH1, efforts whose lessons are directly transferable to any future cardiovascular development program [86].

### 5.3. Soluble Decoy Receptors

An alternative antibody-independent strategy uses soluble, Fc-fused fragments of the Notch extracellular domain (Notch decoys) engineered to selectively sequester either Delta-like ligands or Jagged ligands without themselves activating signaling in the responding cell. Because Jagged- and Delta-like ligand-mediated Notch signaling can produce distinct, sometimes opposing, vascular phenotypes, ligand-selective decoys in principle allow one arm of the pathway to be neutralized while sparing the other, a level of selectivity that is difficult to achieve with conventional pan-Notch γ-secretase inhibition [87]. Although this technology has so far been developed principally for tumor angiogenesis, the underlying design principle—selective neutralization of a single ligand class rather than global receptor blockade—is directly applicable to the endothelial JAG1–NOTCH4 axis implicated in atherogenesis at sites of disturbed flow, and represents one of the more mechanistically elegant, if still preclinical, strategies for future cardiovascular development.

### 5.4. MicroRNA-Based and Epigenetic Strategies

Because several microRNAs sit directly upstream or downstream of Notch pathway components in vascular cells, microRNA mimics or antagomirs offer an indirect route to modulate Notch-dependent phenotypes with potentially greater cell-type selectivity than direct pathway inhibition. Restoring the flow-sensitive microRNA miR-126-5p, which normally maintains endothelial Notch1 activity by repressing the inhibitor Dlk1, reduces atherosclerotic lesion formation and promotes endothelial recovery in murine models [61]. In VSMCs, miR-34a intersects with SIRT1 signaling to regulate proliferation, senescence and osteogenic transdifferentiation; genetic ablation or pharmacological inhibition of miR-34a reduces vascular calcification and senescence markers in murine models [71,72], nominating anti-miR-34a oligonucleotides as a candidate strategy to indirectly normalize Notch3/SIRT1 signaling in the aging, atherosclerotic vessel wall. More broadly, because Notch ligand and receptor expression is subject to extensive post-transcriptional control, combining microRNA-based agents with cell-type-restricted delivery vehicles is an attractive, though still largely preclinical, strategy to achieve the cell-selective Notch modulation that pan-pathway inhibitors cannot provide.

### 5.5. Repurposing Established Cardiovascular Drugs: Statins and Notch-Associated Endothelial Phenotypes

Statins, the cornerstone of lipid-lowering pharmacotherapy in atherosclerotic cardiovascular disease, exert pleiotropic vascular effects beyond LDL-cholesterol reduction, including improvement of endothelial function. Recent work has shown that simvastatin suppresses endothelial-to-mesenchymal transition (EndMT), a process implicated in vascular endothelial dysfunction, in part through epigenetic modulation of a GGTase–RhoA–YAP–SOX9 signaling axis, indicating that at least part of the vasculoprotective, pleiotropic benefit of statin therapy may operate through mechanisms convergent with Notch-associated endothelial phenotypes rather than through cholesterol lowering alone [88]. This crosstalk has a long experimental history: simvastatin was shown more than a decade earlier to promote the differentiation of bone marrow stromal cells into functional endothelial cells specifically via activation of the Notch signaling pathway, an effect abolished by γ-secretase inhibition or Notch1 knockdown [89]. Because statins are already in widespread, well-tolerated clinical use for atherosclerotic cardiovascular disease, and because endothelial-to-mesenchymal transition and Notch-driven endothelial senescence and inflammation converge mechanistically, this crosstalk supports the rationale for combining conventional lipid-lowering therapy with more selective, emerging Notch-directed agents, and raises the possibility that some of the anti-atherosclerotic benefit already achieved with statins in large outcome trials is partly attributable to favorable modulation of vascular Notch signaling.

## 6. Safety Considerations and Translational Challenges

The single most important lesson to emerge from clinical experience with Notch pathway inhibitors, gained almost entirely in oncology, is that this pathway has a narrow therapeutic index in the cardiovascular system. Anti-DLL4 antibodies produce dose- and duration-dependent hypertension, pulmonary hypertension and, in a subset of patients receiving prolonged high-dose exposure, congestive heart failure [43,44,84,85], while pan-Notch γ-secretase inhibitors carry class-wide gastrointestinal and immunological toxicity related to loss of Notch signaling in intestinal and lymphoid progenitor compartments. These toxicities are mechanistically continuous, not incidental, with the pathway’s normal vasculoprotective functions: NOTCH1 signaling maintains endothelial junctional integrity and vascular quiescence [22,30], and profound pharmacological suppression of this signal—rather than the more subtle rebalancing that might be achievable with ligand-selective or vascular bed-restricted agents—predictably destabilizes the endothelium and pulmonary vasculature. Any future Notch-directed therapy for atherosclerosis will therefore need to achieve at least one, and probably several, of the following: (i) selectivity for a single ligand or receptor, for example, JAG1/NOTCH4 rather than pan-Notch [34,87]; (ii) restriction of drug exposure to a specific vascular bed or cell type, through nanoparticle or antibody–drug conjugate delivery; (iii) intermittent or low-dose regimens designed to titrate pathway activity rather than to abolish it; and (iv) combination with existing, well-tolerated background therapy such as statins, which may already provide partial, indirect normalization of Notch-associated endothelial phenotypes and could allow lower doses of a direct Notch-targeted agent to be used [88,89]. In addition, because most direct clinical experience with Notch inhibitors comes from short-term oncology regimens, the long-term cardiovascular safety of chronic, low-intensity Notch modulation—the exposure profile that would be required for a chronic disease such as atherosclerosis—remains essentially untested and will require dedicated long-term toxicology and, eventually, cardiovascular outcome studies.

## 7. Future Perspectives

Several converging lines of investigation suggest realistic paths toward a favorable therapeutic index for Notch-directed atherosclerosis therapy. First, single-cell and spatial transcriptomic mapping of Notch receptor and ligand expression across atherosusceptible and atheroresistant arterial regions should make it possible to define more precisely which receptor–ligand pairs are selectively upregulated at disease-prone sites, refining candidate targets beyond the JAG1–NOTCH4 axis already identified [34]. Second, ligand-selective decoy receptors and next-generation antibody engineering that improve the pharmacokinetic and tissue-distribution profile of anti-DLL4 or anti-JAG1 agents may substantially widen the safety margin relative to first-generation oncology antibodies [86,87]. Third, nanoparticle- or antibody–conjugate-based delivery platforms capable of restricting Notch pathway modulation to macrophages or to endothelium at atherosusceptible sites, rather than achieving systemic pathway blockade, would in principle allow the pro-inflammatory, M1-like-skewing arm of macrophage Notch–RBP-J signaling to be dampened without compromising NOTCH1-dependent endothelial barrier function elsewhere in the vasculature [42,46]. Fourth, microRNA-based agents directed at nodes such as miR-126-5p and miR-34a that intersect with both Notch and senescence or repair pathways may offer a more gradual, combinatorial means of normalizing endothelial and VSMC phenotype than direct pathway blockade [61,71]. Finally, given the mechanistic overlap between statin-mediated modulation of EndMT and Notch pathway biology, prospective biomarker studies nested within existing statin and PCSK9 inhibitor cardiovascular outcome trials could help clarify how much of the clinical benefit of contemporary lipid-lowering therapy is mediated through Notch-dependent endothelial and smooth muscle mechanisms, information that would directly inform the design of combination regimens incorporating future Notch-selective agents.

## 8. Conclusions

The Notch signaling pathway occupies a central, if paradoxical, position in the biology of atherosclerosis: the same core molecular machinery can protect the endothelium from disturbed-flow-induced dysfunction when engaged through NOTCH1, or actively promote lesion formation when engaged through JAG1–NOTCH4 at atherosusceptible sites, drive VSMC dedifferentiation, proliferation and osteogenic transformation through NOTCH1/NOTCH3, and sustain pro-inflammatory macrophage polarization while suppressing reparative macrophage and T-cell programs through Notch–RBP-J signaling. This context dependence explains why global pathway inhibition with γ-secretase inhibitors, although capable of reducing experimental atherosclerosis and neointimal hyperplasia, carries an unfavorable safety profile, and why clinical experience with anti-DLL4 antibodies in oncology has revealed clinically significant cardiopulmonary toxicity. The path toward a clinically viable Notch-directed therapy for atherosclerosis is therefore unlikely to run through pan-pathway blockade, and more likely to depend on ligand-selective antagonists, decoy receptors, microRNA-based modulators, or vascular bed- and cell type-restricted delivery platforms capable of rebalancing, rather than abolishing, Notch signaling within the diseased artery wall.

## Figures and Tables

**Table 1:** Core Notch pathway components relevant to vascular biology and atherosclerosis.

Component	Type	Predominant Vascular Expression	Role in Atherosclerosis-Relevant Vascular Biology
NOTCH1	Receptor	Arterial endothelium; VSMC	Mechanosensor; atheroprotective under laminar shear stress; drives VSMC dedifferentiation when aberrantly activated [30]
NOTCH2	Receptor	Endothelial cells	Downregulated by TNF-α/IL-1β; contributes to endothelial dysfunction [31]
NOTCH3	Receptor	VSMC/pericytes	Required for arterial mural cell differentiation and survival; mutated in CADASIL; mediates osteogenic transdifferentiation [11,13,32,33]
NOTCH4	Receptor	Endothelial cells	Paired with JAG1 at low-oscillatory-shear, atherosusceptible sites; pro-atherogenic; also supports endothelial quiescence when intact [34,35]
JAG1	Ligand	Endothelium; antigen-presenting cells; mural cells	Cis-inhibits NOTCH1; trans-activates NOTCH4; promotes leukocyte recruitment; non-canonical VEGFR2/ERK mechanotransducer; maintains NOTCH3 in mural cells [23,34,36,37]
JAG2	Ligand	Endothelium; VSMC	Developmental vascular roles; less well characterized in adult atherosclerosis
DLL1	Ligand	Endothelium; antigen-presenting cells	Promotes pro-inflammatory (Th1/Th17) T-cell differentiation [38,39]
DLL4	Ligand	Arterial/tip endothelial cells; macrophages	Upregulated by IL-1β, TNF-α, hyperlipidemia; drives macrophage M1-like polarization and suppresses M2-like; target of anti-DLL4 antibodies [40,41,42,43,44]
RBP-J (CSL/CBF1)	Transcription factor	Ubiquitous	Canonical nuclear effector of NICD; induces IRF8-dependent M1-like macrophage program; also supports selected M2-like genes [45,46,47,48]
HES1/HES5, HEY1/HEY2	Target genes	Endothelium; VSMC; valve interstitial cells	Downstream effectors of NICD–RBP-J; repress contractile/osteogenic genes; Hey1/Hey2 required for valve EMT [21,49]
γ-Secretase complex	Protease complex	Ubiquitous	Catalyzes S3 cleavage releasing NICD from all Notch receptors; principal target of GSIs (e.g., DAPT) [5,50,51]

**Table 2:** In vivo effects of experimental Notch pathway manipulation on atherosclerosis and related vascular remodeling.

Manipulation	Model/System	Effect on Atherosclerosis/Lesion Phenotype	Ref.
Endothelial-specific Notch1 deletion or haploinsufficiency	ApoE−/− or hypercholesterolemic mice	Significantly increased lesion burden in the descending aorta and aortic sinus; loss of junctional integrity and increased endothelial proliferation at disturbed-flow sites	[30,53]
Endothelial-specific Jag1 deletion	Murine and porcine arteries	Significantly reduced lesion burden specifically at low-oscillatory-shear, atherosusceptible sites, with no effect at protected sites	[34]
Systemic Dll4 neutralizing antibody	Hyperlipidemic LDL receptor-deficient mice	Attenuated atherosclerotic lesion formation together with amelioration of associated metabolic derangements (adipose inflammation, insulin resistance)	[41]
Macrophage-derived Dll4 (loss-of-function)	Murine vein-graft model	Reduced vein-graft lesion development, supporting a pro-atherogenic role for macrophage-derived Dll4 distinct from its endothelial source	[78]
Macrophage-specific Notch1 deletion	Murine atherosclerosis model	Shifted plaque macrophages toward an M2-like anti-inflammatory profile via the PI3K-oxidative stress axis, consistent with reduced lesion inflammation	[20]
Pan-Notch γ-secretase inhibition (systemic GSI)	ApoE−/− mice, diet-induced atherosclerosis	Reduced diet-induced atherosclerotic lesion formation, demonstrating net atheroprotection despite non-selective pathway blockade over the exposure durations tested	[51]
γ-Secretase inhibition with DAPT	Rat vein-graft model of restenosis	Restored contractile marker gene expression, suppressed VSMC proliferation and reduced intimal hyperplasia	[50]
Notch1 inhibition (siRNA or DAPT) combined with induced low shear stress	ApoE−/− mice, carotid shear-stress modifier model	DAPT reduced low-shear-stress-induced plaque formation and ICAM-1 expression via suppression of NF-κB activation, directly linking flow-driven Notch1 induction to lesion formation in vivo	[83]

**Table 3:** Notch-targeted therapeutic strategies for atherosclerosis.

Strategy	Representative Agent(s)	Mechanism	Stage of Development	Key Limitations/Safety Signals
Pan-Notch γ-secretase inhibition	DAPT and related GSIs	Blocks S3 cleavage of all four Notch receptors	Preclinical (ApoE−/− mice; vein-graft/restenosis models) [50,51]	Non-selective (additional γ-secretase substrates); gastrointestinal and lymphoid toxicity limit chronic use
Anti-DLL4 monoclonal antibodies	Demcizumab (OMP-21M18)	Blocks DLL4–NOTCH1 ligand engagement	Phase I/Ib oncology trials [43,44,84]	Hypertension, pulmonary hypertension, reversible heart failure with prolonged dosing [85]
Notch2/3-selective antibodies	Tarextumab	Selective receptor antagonism sparing NOTCH1	Phase I/II oncology [86]	Narrower target engagement; chronic cardiovascular safety not established
Ligand-selective decoy receptors	Engineered Notch-Fc decoys (Dll4- or Jagged-selective)	Sequester a specific ligand class without activating the receiving cell	Preclinical (tumor angiogenesis models) [87]	Not yet tested in atherosclerosis models; delivery/PK optimization needed
MicroRNA-based modulation	miR-126-5p mimics; anti-miR-34a oligonucleotides	Restores endothelial Notch1/Dlk1 balance; normalizes Notch3-associated VSMC senescence/calcification	Preclinical (murine atherosclerosis and vascular calcification models) [61,71,72]	Vascular cell-type-specific delivery remains challenging
Drug repurposing	Statins (e.g., simvastatin)	Suppresses EndMT via GGTase–RhoA–YAP–SOX9 axis; activates Notch-dependent endothelial differentiation	Established clinical use; mechanistic Notch/EndMT link described [88,89]	Effect on Notch signaling is indirect and pleiotropic; Notch-specific contribution to clinical benefit unclear

## Data Availability

No new data were created or analyzed in this study. Data sharing is not applicable to this article.
